# Novel loss-of-function *SPAG*17 homozygous variant segregated in a family with severe asthenozoospermia: upgrading gene-disease validity to strong

**DOI:** 10.3389/fendo.2026.1754106

**Published:** 2026-02-19

**Authors:** Li Wang, Jinli Li, Ling Huang, Jialing Wang, Li Zhou, Li Ding, Jia Li, Qinghua Zhang, Junyu Zhang, Guangmei Xie

**Affiliations:** 1 Gansu Provincial Maternity and Child-care Hospital(Gansu Provincial Central Hospital), The Second Reproductive Medicine Center, Lanzhou, China; 2 Reproductive Medicine Center, Shanghai First Maternity and Infant Hospital, School of Medicine, Tongji University, Shanghai, China; 3 Medical Genetics Center, Gansu Provincial Maternity and Child-care Hospital(Gansu Provincial Central Hospital), Lanzhou, Gansu, China; 4 The First Clinical Medical College of Gansu University of Chinese Medicine, Lanzhou, Gansu, China

**Keywords:** gene-disease validity, male infertility, severe asthenozoospermia, *SPAG17*, sperm ultrastructural abnormalities

## Abstract

**Background:**

Severe asthenozoospermia is a significant cause of male infertility, commonly associated with genetic defects affecting sperm motility. However, the specific genetic contributors remain underexplored.

**Objective:**

This study aimed to identify a genetic variant responsible for severe asthenozoospermia in two siblings and to evaluate the clinical validity of the gene-disease relationship between *SPAG*17 and this condition.

**Methods:**

Whole exome sequencing (WES) was performed on two siblings diagnosed with severe asthenozoospermia. Sperm motility and morphology were assessed through standard semen analysis and transmission electron microscopy (TEM). The gene-disease validity was evaluated using the ClinGen Gene–Disease Validity SOP, incorporating both genetic and experimental evidence.

**Results:**

A novel homozygous nonsense variant in *SPAG*17 (NM_206996.4: c.2188C>T; p.Q730*) was identified in both affected siblings. Semen analysis revealed significantly reduced sperm motility and abnormal sperm morphology, including malformed flagella. TEM showed severe axonemal defects, such as absent central-pair microtubules and disorganized axonemal structures. The gene-disease validity between *SPAG*17 and severe asthenozoospermia was upgraded to “Strong”, with a cumulative score of 13.4 points based on genetic (9.4 points) and experimental (4 points) evidence.

**Conclusion:**

We identified a novel homozygous nonsense variant in *SPAG*17 in two siblings with severe asthenozoospermia, emphasizing its critical role in sperm motility and male fertility. The upgraded “Strong” gene-disease validity strengthens *SPAG*17’s clinical utility for genetic diagnostics and counseling.

## 1 Introduction

Sperm motility is a crucial determinant of fertility, which represents a fundamental feature of flagellate spermatozoa and playing a vital role in the fertilization process (1). Asthenozoospermia is a major cause of male infertility, affecting approximately 19% of infertile patients. It is defined by a progressive motility (PR) rate of spermatozoa below 32% (2). Asthenozoospermia has been attributed to various factors, including genetic causes, lifestyle, environmental pollutants, prolonged sexual abstinence, partial blockage of the seminal tract, varicocele, and infections (3–6). Although genetic deficiencies are significant causal factors (7), the specific genetic contributors to this condition remain largely unexplored.

*SPAG17* encodes a protein located in the axoneme central pair complex of motile cilia and flagella (8). Previous studies in chlamydomonas have demonstrated that PF6, the ortholog of the mammalian *SPAG*17 protein, is critical for flagellar motility (9). *Spag*17 knockout mice exhibit infertility due to a severe defect in spermatogenesis. Histological analysis of testis sections from mutant mice revealed seminiferous tubules with spermatogenesis arrested at the spermatid stage. The few sperm retrieved from the cauda epididymis were immotile and exhibited abnormalities morphology (8). According to reports, several male cases with infertility carry homozygous variants in the *SPAG17* gene (10–12). These findings collectively underscore the critical role of *SPAG*17 in sperm development, motility, and male fertility.

Despite this accumulating evidence, a formal evaluation of the clinical validity of the gene-disease association between *SPAG*17 and severe asthenozoospermia has not been thoroughly performed. Evaluating the clinical validity of the gene-disease relationship is essential for variant classification and clinical interpretation (13). The Clinical Genome Resource (ClinGen) has developed a semiquantitative points-based framework to assign clinical validity classifications to gene–disease relationships (14, 15). Clinical validity is categorized as definitive (12–18 points, replicated over time), strong (12–18 points), moderate (7–11 points), limited (0.1–6 points), no known disease relationship (0 points), disputed (contradictory evidence), or refuted (contradictory evidence outweighs supportive evidence). The ACMG recommends that variants in moderate genes should generally not be classified higher than Likely Pathogenic, and variants in genes with poorly understood disease-gene relationships (Limited, Disputed, Refuted) should not be classified higher than variants of uncertain significance (16).

In this study, we identified a novel homozygous nonsense variant in *SPAG17* in two siblings with severe asthenozoospermia significantly contributes to understanding the genetic basis of this condition. The detailed semen analysis and ultrastructural studies revealed profound abnormalities in sperm morphology and flagellar structure, further supporting the involvement of *SPAG*17 in male infertility. This study, in conjunction with previous reports and emerging reports, substantially enhances the evidence for the gene-disease relationship between *SPAG*17 and severe asthenozoospermia, elevating the association to a strong strength according to the ClinGen Gene–Disease Validity SOP. These findings underscore the importance of *SPAG*17 in spermatogenesis and male fertility, providing valuable insights into potential diagnostic and therapeutic approaches for affected individuals.

## 2 Methods

### 2.1 Patients and ethics statement

This study received ethical approval from the Gansu Provincial Maternal and Child Health Hospital (Gansu Provincial Central Hospital), and informed consent was obtained from each participant. The brothers with primary infertility and their mother were recruited from the Gansu Provincial Maternal and Child Health Hospital (Gansu Provincial Central Hospital).

### 2.2 Whole exome sequencing, variant calling, and sequence variant validation

Genomic DNA was isolated from peripheral blood lymphocytes of participants using QIAamp DNA Blood Kits (Qiagen, Hilden, Germany). Library preparation and exome enrichment were conducted using SureSelect Human All Exon V6 (Agilent Technologies, Santa Clara, CA, USA). The prepared libraries were sequenced using the Illumina NovaSeq^®^ 6000 system (Illumina, San Diego, CA, USA). The variants were filtered and prioritized using TGex (https://fa.shanyint.com/, accessed 4 January 2025) (17). The gene variants detected by WES were validated by Sanger sequencing, using the following primers for amplification and sequencing: *SPAG*17-c2188-F=CCGAGAACCTTCAGATCCTAGTC and *SPAG*17-c2188-R=AGGGTTGAAATAAAGAGAACTTATGG.

### 2.3 Literature review and gene–disease clinical validity curation

A comprehensive literature search was conducted using PubMed and Google Scholar to identify publications on the relationship between *SPAG*17 and male infertility in humans and animal models. Only English-language manuscripts and abstracts were considered. Cases reporting deleterious *SPAG*17 variants associated with male infertility due to asthenozoospermia were reviewed. The strength of the gene–disease association between *SPAG*17 and autosomal recessive severe asthenozoospermia was curated following the ClinGen Gene–Disease Validity SOP, version 11 (18).

### 2.4 Semen parameters and sperm morphology analysis

Semen samples were collected by masturbation and incubated at 37 °C for 30 minutes to achieve liquefaction. Routine semen analysis (volume, sperm concentration, and motility) was performed using the Sperm Class Analyzer CASA System (SCA, Spain) following World Health Organization guidelines (5th Edition) (19). Semen samples from subjects were washed with PBS, fixed in 4% paraformaldehyde (PFA), and stained with Mayer’s hematoxylin and a 1% eosin Y solution (FUJIFILM WakoPure Chemical). Sperm morphology was assessed by examining at least 130 sperm from each sample.

### 2.5 Transmission electron microscopy

Spermatozoa were immersed in a 2.5% glutaraldehyde solution, washed three times with 0.1 mol/L phosphate buffer (PB, pH 7.2), and postfixed with 1% osmium tetroxide in 0.1 mol/L PB for 1–1.5 hours at 4 °C. Dehydration was performed sequentially using ethanol solutions at concentrations of 50%, 70%, 80%, 95%, and 100%, followed by 100% acetone. Infiltration was then carried out by immersing the samples overnight at 37 °C in a 1:1 mixture of acetone and SPI-Chem resin (containing dodecenyl succinic anhydride, N-methylacetamide, SPI-Pon 812, and DMP-30). After infiltration and embedding in Epon 812 resin, ultrathin sections were stained with uranyl acetate and lead citrate. These sections were then examined and photographed using a TEM (TECNAI-10, Philips) operating at an accelerating voltage of 80 kV.

### 2.6 Normal controls

For comparative analyses in semen morphology and ultrastructural studies, normal control samples were obtained from three healthy fertile volunteers with normal semen parameters (progressive motility > 40%, normal morphology > 4%) and no history of reproductive disorders. Control samples were processed identically and simultaneously with patient samples under the same laboratory conditions, including fixation, staining, and TEM preparation, to minimize batch effects and pre-analytical variability.

## 3 Result

### 3.1 Severe asthenozoospermia and ultrastructural abnormalities in infertile two siblings

**Figure 1:**
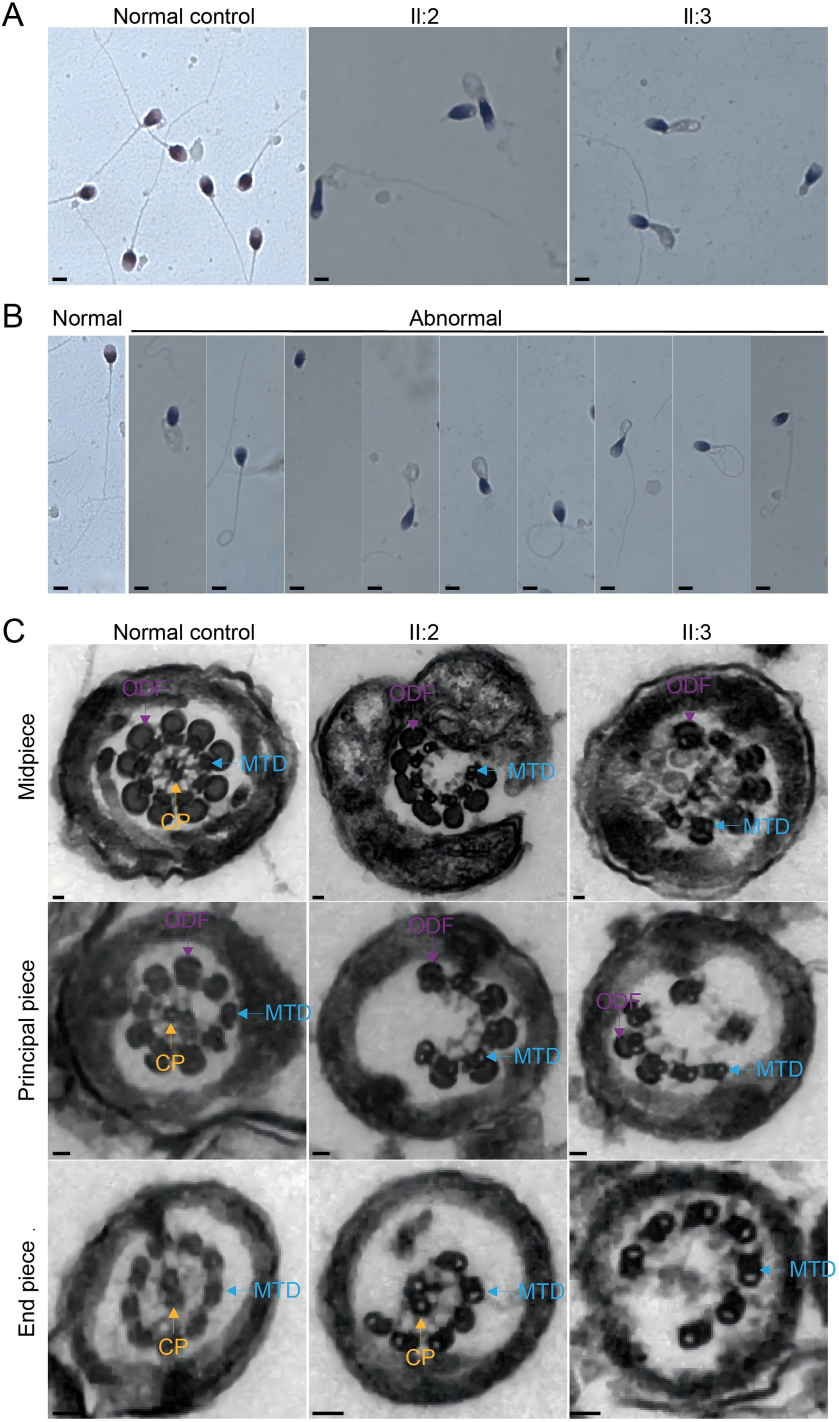
Morphological and ultrastructural analysis of spermatozoa in normal controls and individuals with bi-allelic *SPAG17* variants. **(A)** Morphology of the spermatozoa from normal control and men harboring bi-allelic *SPAG17* variants (scale bars, 15 μm). **(B)** Papanicolaou staining revealed sperm with normal morphology and various abnormal morphologies (scale bars, 15 μm). **(C)** Representative TEM micrographs display cross-sections of the midpiece, principal piece, and end piece of sperm flagella from normal controls and individuals with bi-allelic *SPAG17* variants. The axoneme structure, along with its accessory components illustrated in the diagram, includes peripheral microtubule doublets (DMT), central pairs (CP), and outer dense fibers (ODF) (scale bars, 150 nm).

The patient and his sibling, with primary infertility durations of 10 and 3 years, respectively, both exhibit severe asthenozoospermia. Sperm motility and progressive motility of them were both significantly below the normal reference values (**Table 1**). Hematoxylin and eosin (H&E) staining was utilized to evaluate sperm morphology. The sperm from the two patients both exhibited a reduced proportion of morphologically normal sperm and an increased prevalence of abnormalities in flagella, including absent, short, curly, angular, and irregularly calibrated flagella (**Figure 1**, **Table 1**). Additionally, transmission electron microscopy (TEM) was used to analyze the ultrastructure of spermatozoa from the two siblings. Strikingly, compared to the regular “9 + 2” axonemal arrangement observed in sperm flagella from normal controls, the spermatozoa from the two patients exhibited absent or disorganized central-pair microtubules (CPs) and irregular or absent outer dense fibers (ODFs) and microtubule doublets (MTDs) in the flagellar midpiece. Additionally, the principal piece showed a loss of most axonemal microtubules, which were irregularly arranged, and the peripheral MTDs or CPs were absent in the end piece.

**Table 1:** Clinical assessment of patients carrying *SPAG17* variants.

Information	II:2	II:3	Reference values
Genotype	MT/MT	MT/MT	
Age (years)	37	34	
Years of marriage	10	3	
BMI	23.67	19.59	
Semen parameters			
Semen volume (ml)	3.47±1.19	1.86±0.21	>1.5
Semen pH	Alkaline	Alkaline	Alkaline
Sperm concentration (10^6^/ml, Mean ± SD)	45.7±23.11	77.02±39.40	>15
Motile sperm (%, Mean ± SD)	3.33±1.15	3.20±1.79	>40
Progressively motile sperm (%, Mean ± SD)	2.33±0.58	0.8±0.45	>32
Sperm flagellar morphology			
Absent flagella (%)	22.79	10.07	<5.0
Short flagella (%)	15.44	11.51	<1.0
Coiled flagella (%)	23.53	22.3	<17.0
Angulation (%)	16.91	20.14	<13.0
Irregular caliber (%)	13.23	27.34	<2.0
Normal flagella (%)	8.09	8.63	>23.0

Additionally, transmission electron microscopy (TEM) was used to analyze the ultrastructure of spermatozoa from the two siblings. A total of 60 cross-sectional flagellar images per individual were evaluated. Compared to the regular “9 + 2” axonemal arrangement observed in controls, patient spermatozoa exhibited absent central-pair microtubules in 82% of cross-sections, disorganized outer dense fibers in 75%, and irregular microtubule doublets in 68%. Representative images are shown in **Figure 1C**.

### 3.2 Identification of a homozygous loss-of-function variant in *SPAG*17 in two siblings affected by severe asthenozoospermia.

**Figure 2:**
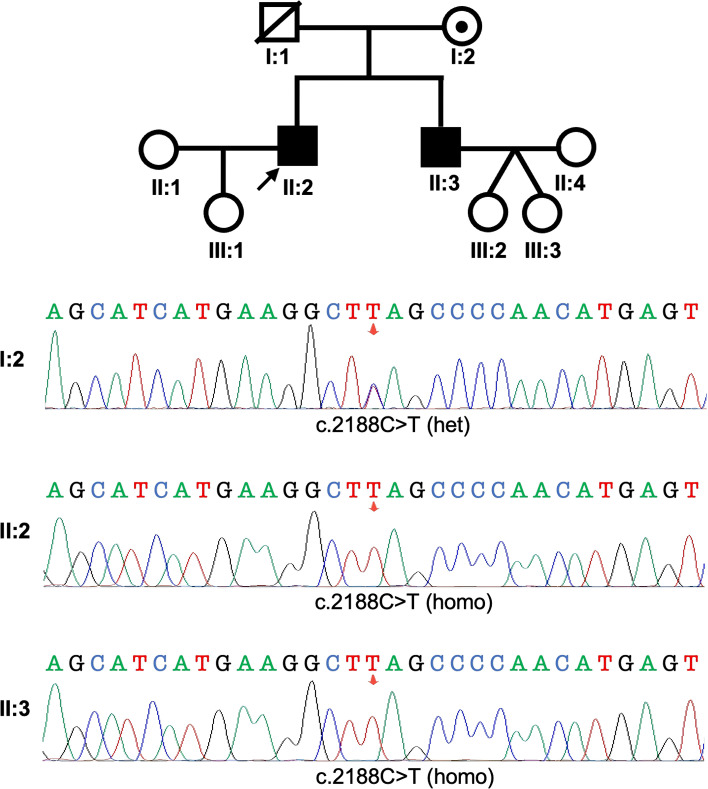
Identification of a homozygous *SPAG*17 variant in two infertile siblings with severe asthenozoospermia. Top: Pedigree of a family with two infertile males (II:2 and II:3), highlighting the proband indicated by an arrow. Bottom: Sanger sequencing chromatograms are shown for the tested individuals. The affected males harbor a homozygous loss-of-function variant in *SPAG*17.

Prior to referral, both individuals had underwent unsuccessful intracytoplasmic sperm injection (ICSI) cycles at an external clinic. Subsequent diagnostic genetic testing identified an identical homozygous nonsense variant in *SPAG*17 (NM_206996.4: c.2188C>T; p.Q730*) in both siblings, resulting in a premature stop codon. Sanger sequencing confirmed homozygosity for the *SPAG*17 variant in both patients and revealed that their mother was heterozygous for the same variant (**Figure 2**), consistent with autosomal recessive inheritance. The father, now deceased, could not be tested due to the unavailability of DNA. Following another ICSI cycle combined with assisted oocyte activation (AOA), both individuals achieved live births: II:2 had a daughter, and II:3 had twin daughters.

### 3.3 The addition of this case upgrades the gene–disease validity between *SPAG*17 and autosomal recessive severe asthenozoospermia from “Moderate” to “Strong”

The identification of a novel homozygous loss-of-function variant in *SPAG*17 associated with severe asthenozoospermia prompted a formal evaluation of the gene-disease validity between *SPAG*17 and severe asthenozoospermia. The curation considered both genetic and experimental evidence. The current cases contributed significant genetic evidence, with 3 points awarded based on the identification of a predicted null variant in an autosomal recessive condition. This was combined with evidence from 4 previous report male cases with infertility harboring two homozygous missense variants and two homozygous loss of function variants in *SPAG*17 (**Table 2**) (10–12). Collectively, the cumulative genetic evidence scored 9.4 out of a total of 12 points (**Table 3**) (15, 18).

**Table 2:** Cases with homozygous variants in *SPAG17* and severe asthenozoospermia.

Individual	Age at diagnosis	Variant in *SPAG17*	Exon	Clinical diagnosis	Inheritance	Reference
II:5	29	NM_206996.4:c.4343G>A p.(Arg1448Gln)	30	Severe asthenozoospermia	Homozygous	(11)
II:7	29	NM_206996.4:c.4343G>A p.(Arg1448Gln)	30	Severe asthenozoospermia	Homozygous	(11)
(Patient 1)IV:1	30	NM_206996.2:c.829+1G>T p.(Asp212_Glu276del)	6	multiple morphological abnormalities of the flagella	Homozygous	(10)
(Patient 2)IV:3	25	NM_206996.2:c.829+1G>T p.(Asp212_Glu276del)	6	multiple morphological abnormalities of the flagella	Homozygous	(10)
(Patient 3)IV:1	42	NM_206996.4:c.2120del p.(Leu707Ter)	15	multiple morphological abnormalities of the flagella	Homozygous	(10)
(Patient 4)IV:4	33	NM_206996.4:c.2120del p.(Leu707Ter)	15	multiple morphological abnormalities of the flagella	Homozygous	(10)
Patient 1	35	NM_206996.4:c.4511A>G p.(Asn1504Ser)	−	oligoasthenoteratozoospermia	Homozygous	(12)
Patient 1	34	NM_206996.4:c.2188C>T p.(Gln730Ter)	15	Severe asthenozoospermia	Homozygous	This study
Patient 2	37	NM_206996.4:c.2188C>T p.(Gln730Ter)	15	Severe asthenozoospermia	Homozygous	This study

II:5 and II:7 are twins.

Patient 1 and patient 2 are brothers.

(Patient 1)IV:1 and (Patient 2)IV:3 are from one consanguineous Pakistani families.

(Patient 3)IV:1 and (Patient 4)IV:3 are from another consanguineous Pakistani families.

**Table 3:** Genetic evidence summary matrix for evaluating the strength of the gene-disease validity between *SPAG17* and autosomal recessive male infertility.

Genetic evidence: case-level data					
Evidence type	Case information	Suggested upgrades		Points given	References/Notes
		Functional data	*De Novo*		
Variant Evidence: Autosomal Dominant*2	Predicted or proven null variant (default 1.5 points, scoring range 0-3 points per variant)	+0.5 points	+0.5 points	9.0 points	Two homozygous frameshift variants in *SPAG17*, resulting in premature termination codons, have been identified in infertile men (see **Table 2** for a case of a pair of brothers from this study and a case of a pair of brothers from previous research (10)). These two variants are predicted to induce nonsense-mediated transcript decay, as the premature termination codons are located more than 50 bp upstream of the final exon-exon junction (12). A homozygous canonical splice site in *SPAG17* has been identified in infertile men (see **Table 2** for a case of a pair of brothers from previous research (10)), The variant led to significantly reduced SPAG17 mRNA levels and no detectable SPAG17 protein in the spermatozoa of patient. All variants were assigned the default of 1.5 points.
	Other variant type with some evidence of gene impact (default 0.1 points, scoring range 0-1.5 points per variant)	+0.4 points	+0.4 points	0.4 points	Two homozygous missense variants in *SPAG17*, have been identified in infertile men (see **Table 2** for a pair of twins case from previous research (11) and another case from previous research (12)). These variants are assigned a default score of 0.1 points.
Segregation Evidence	Evidence of segregation in one or more families (scoring range 0-3 points)			0	No evidence available
Genetic Evidence: case-control data					
Case-Control Study Type	Case-Control Quality Criteria	Suggested Points/Study		Points Given	References/Notes
Single Variant Analysis	•Variant detection methodology •Power •bias and confounding factors •Statistical significance	0-6 points		0	No evidence available
Aggregate Variant Analysis		0-6 points		0	No evidence available
Total genetic evidence points				9.4	

Clinical validity of gene-disease validity was curated using the Clinical Genome Resource (ClinGen) framework, version 11 (18).

The correlation between *SPAG*17 and severe asthenozoospermia is also supported by experimental evidence (**Table 4**). *SPAG*17 is crucial for the structure and function of motile cilia (20) and facilitates the translocation of protamines from the cytoplasm to the nucleus, a key process in sperm production (21). *SPAG*17 is highly expressed in testicular tissue, where spermatogenesis occurs (22). In *SPAG*17 knockout mice, primary cilia in chondrocytes, osteoblasts, and embryonic fibroblasts (MEFs) were shorter, and fewer cells exhibited primary cilia compared to wild-type mice (23). *Spag*17 knockout mice are infertile due to a severe spermatogenesis defect, with spermatogenesis arrested at the spermatid stage. Sperm retrieved from the cauda epididymis were immotile and displayed abnormal tail and head morphology (8). These data underscore the critical role of *SPAG*17 in male reproductive biology, with a cumulative evidence score of 4 out of 6 allowable points according to the ClinGen Gene–Disease Validity SOP (**Table 4**) (13, 18).

**Table 4:** Experimental evidence summary matrix for evaluating the strength of the gene-disease validity between *SPAG17* and male infertility.

Experimental evidence					
Evidence category	Evidence type	Suggested points		Points given	References/Notes
		Default	Range		
Function	Biochemical function	0.5	0-2	1.0	*SPAG17* is essential for the structure and function of motile cilia (20). *SPAG17* facilitates the translocation of protamines from the cytoplasm to the nucleus, a process essential for sperm production (21).
	Protein interaction	0.5	0-2	0	No evidence available
	Expression	0.5	0-2	0.5	SPAG17 is highly expressed in testis tissue, where spermatogenesis takes place (22).
Functional Alteration	Patient cells	1	0-2	0	No evidence available
	Non-patient cells	0.5	0-1	0.5	Primary cilia in chondrocytes, osteoblasts, and embryonic fibroblasts (MEFs) from knockout mice were shorter, and fewer cells had primary cilia compared to those from wild-type mice (23).
Models	Non-human model organism	2	0-4	2	Spag17 knockout mice are infertile due to a severe defect in spermatogenesis, with spermatogenesis arrested at the spermatid stage. The few sperm retrieved from the cauda epididymis were immotile and exhibited abnormal tail and head morphology (8).
	Cell culture model	1	0-2	0	No evidence available
Rescue	Rescue in human	2	0-4	0	No evidence available
	Rescue in non-human model organism	2	0-4	0	No evidence available
	Rescue in cell culture model	1	0-2	0	No evidence available
	Rescue in patient cells	1	0-2	0	No evidence available
Total experimental evidence points				4.0	

Clinical validity of gene-disease validity was curated using the Clinical Genome Resource (ClinGen) framework, version 11 (18).

Combining the genetic (9.4 points) and experimental (4 points) evidence resulted in a total score of 13.4 points. According to the ClinGen Gene–Disease Validity SOP, the 3 points contributed by our cases upgraded the gene–disease validity between *SPAG*17 and autosomal recessive severe asthenozoospermia from “Moderate” (requiring 7–11 points) to “Strong” (requiring 12–18 points).

## 4 Discussion

The identification of a novel homozygous nonsense variant in *SPAG*17 (NM_206996.4:c.2188C>T; p.Q730*) in these two siblings with severe asthenozoospermia significantly strengthens the genetic evidence implicating *SPAG*17 in this specific form of male infertility. Our finding, along with the previously reported homozygous missense variant (p. R1448Q) in the *SPAG*17 gene in twins with severe asthenozoospermia (11), and the more recently identified homozygous variants (c.4511A>G, p.Asn1504Ser; c.829 + 1G>T, p.Asp212_Glu276del; c.2120del, p.Leu707*) associated with oligoasthenoteratozoospermia (OAT) and MMAF phenotypes (10, 12), provides genetic evidence of *SPAG*17’s crucial role in sperm motility and male infertility. The sperm motility, morphology, and ultrastructure observed in the patients analyzed in this study align closely with findings from prior animal model research on *SPAG*17. Sperm motility analysis revealed severely reduced progressive motility, consistent with the immotile sperm phenotype reported in *Spag*17 knockout mice (8). Morphological evaluation demonstrated abnormalities, such as absent, short, and irregularly shaped flagella, consistent with defective sperm tails in knockout mice (8). Additionally, ultrastructural examination demonstrated disruptions in the “9 + 2” axonemal arrangement, including disorganized or absent central-pair microtubules, outer dense fibers, and microtubule doublets, consistent with flagellar defects reported in *Spag*17 knockout animal models (8). In addition to animal knockout models, other experimental evidence further supports the link between *SPAG*17 and severe asthenozoospermia. *SPAG*17 is essential for the structure and function of motile cilia and for the translocation of protamines during sperm production (20, 21). It is highly expressed in testicular tissue, where spermatogenesis occurs (22). The consistent findings of significantly reduced sperm motility and the array of flagellar abnormalities, both at the microscopic and ultrastructural levels, directly mirror the phenotypes observed in *Spag*17 knockout animal models, further reinforcing the functional consequence of *SPAG*17 deficiency in humans.

The central significance of our study lies in its impact on the formal evaluation of the gene-disease validity between *SPAG*17 and severe asthenozoospermia. Prior to this study, while evidence from *Spag*17 knockout mouse models and human case report (8, 10–12) suggested a link, the overall gene-disease validity, as assessed by the rigorous ClinGen Gene-Disease Validity SOP, was classified as “Moderate”(10.4 points). Our identification of this novel, loss-of-function homozygous variant in two affected siblings provided the critical additional genetic evidence needed to strengthen the association. Our cases, contributing a significant 3 points to the genetic evidence score within the ClinGen framework, effectively upgraded the overall evidence beyond the threshold required for “Strong”(13.4 points).

This upgrade from “Moderate” to “Strong” has significant implications for the clinical management of male infertility. Firstly, it provides more substantial support for including *SPAG17* in diagnostic gene panels for asthenozoospermia (16), enhancing the precision of genetic testing and improving the likelihood of identifying the underlying genetic cause in similar cases. Secondly, the “Strong” classification has a direct impact on *SPAG17* variant in clinical settings. Variants in genes with “Moderate” validity could be classified as “Likely Pathogenic”, in contrast, for genes with a disease association categorized as definitive or strong, variants can be classified as high as pathogenic (16). This shift provides more actionable information for patient management and reproductive planning.

Notably, the clinical management of the affected siblings in our study provides further insight into the multifaceted role of *SPAG17*. Both individuals underwent ICSI combined with assisted oocyte activation (AOA), suggesting potential fertilization impairment beyond mere motility defects. *SPAG17* is known to facilitate nuclear translocation of protamines during spermiogenesis, and its deficiency may lead to chromatin abnormalities and impaired sperm-derived oocyte activation capacity. This aligns with emerging evidence that flagellar structural proteins can also influence sperm nuclear integrity and centriolar function. Therefore, AOA may address not only the overt motility barrier but also underlying chromatin or activation deficiencies in SPAG17-deficient sperm.

In summary, we identified a novel homozygous loss-of-function variant in *SPAG*17 in two affected siblings. Semen analysis and ultrastructural studies have revealed a significant reduction in sperm motility and severe morphological abnormalities in affected patients. Combined with previous genetic and experimental evidence, these findings upgrade the gene-disease validity for *SPAG*17-related autosomal recessive severe asthenozoospermia to “Strong”. Our findings highlight the critical role of *SPAG*17 in male fertility and have significant clinical implications, supporting the inclusion of *SPAG*17 in male infertility screening gene panels, enabling more reliable variant classification, and ultimately enabling more precise genetic counseling and therapeutic strategies for affected individuals.

### 4.1 Study limitations and future perspectives

This study has certain limitations. Most notably, due to the scarcity and complete consumption of patient sperm samples for essential diagnostic and ultrastructural analyses (e.g., TEM), we were unable to perform direct protein-level validation, such as immunofluorescence or Western blotting, to confirm the absence of the *SPAG17* protein. While the identification of a homozygous nonsense variant provides strong genetic evidence for a loss-of-function mechanism, future studies with access to relevant samples are encouraged to validate these findings at the protein level. Additionally, the functional link between *SPAG17* deficiency and the observed need for assisted oocyte activation warrants further mechanistic investigation.

## Data Availability

The original contributions presented in the study are included in the article/supplementary material. Further inquiries can be directed to the corresponding authors.
